# Laparoscopic-Assisted Colorectal Resection Can Reduce the Inhibition of Immune Function Compared with Conventional Open Surgery: A Retrospective Clinical Study

**DOI:** 10.3390/jcm12062320

**Published:** 2023-03-16

**Authors:** Bo Shi, Qingliang Tai, Junjie Chen, Xinyu Shi, Guoliang Chen, Huihui Yao, Xiuwei Mi, Jinbing Sun, Guoqiang Zhou, Wen Gu, Songbing He

**Affiliations:** 1 Department of General Surgery, The First Affiliated Hospital of Soochow University, Suzhou 215005, China; 2 Department of General Surgery, Suzhou Ninth Hospital Affiliated to Soochow University, Suzhou 215000, China; 3 Department of General Surgery, Changshu Hospital Affiliated to Soochow University, First People’s Hospital of Changshu City, Changshu 215501, China; 4 Department of Gastrointestinal Surgery, Changshu No. 2 Hospital, Changshu 215123, China

**Keywords:** laparoscopic operation, colorectal cancer, immune function, surgical trauma, T-lymphocyte subsets

## Abstract

Background: Immune function is an important indicator for assessing postoperative recovery and long-term survival in patients with malignancy, and laparoscopic surgery is thought to have a less suppressive effect on the immune response than open surgery. This study aimed to investigate this effect in a retrospective clinical study. Methods: In this retrospective clinical study, we enrolled 63 patients with colorectal cancer in the Department of General Surgery of the First Affiliated Hospital of Soochow University and assessed the changes in their postoperative immune function by measuring CD3^+^T, CD4^+^T, CD8^+^T lymphocytes, and CD4^+^/CD8^+^ ratio. Results: Compared with open surgery, laparoscopic colorectal surgery was effective in improving the postoperative decline in immune function. We determined that the number of CD4^+^, CD8^+^T lymphocytes, and the CD4^+^/CD8^+^ ratio was not significantly reduced in the laparoscopic group. Conclusion: Laparoscopic-assisted colorectal resection can reduce the inhibition of immune functions compared with conventional open surgery.

## 1. Introduction

Colorectal cancer is one of the most common malignancies worldwide and the leading cause of cancer-related mortality [1]. In recent years, it has been determined that the occurrence of malignant tumors is related to the tumor microenvironment [2,3]. T cell infiltration, activation, and effector functions are inhibited by tumors when immune evasion occurs, which causes the body to increase immune tolerance, leading to ineffective immune response and tumor progression [4,5]. T cells can be divided into two subpopulations, CD3^+^CD4^+^ and CD3^+^CD8^+^ T cells, according to different surface antigens, and the balance between them is an important insurance for maintaining normal immune system work, and the ratio of the two is an important indicator to assess immune function [6,7]. In a recent study, CD3^+^ T lymphocytes and CD4^+^/CD8^+^ ratio levels on the second postoperative day were determined to be higher in patients who underwent laparoscopic-assisted natural orifice specimen extraction than in the conventional laparoscopic-assisted radical resection group, thus confirming the early safety after laparoscopic-assisted natural orifice specimen extraction [8]; Gang Wang et al. showed that fast-track surgery had better protection of patients’ immune function postoperatively compared to laparoscopic surgery, with less impact on CD3^+^,CD4^+^T lymphocytes, CD4^+^/CD8^+^ ratio and fewer perioperative complications [9]. Hence, detecting changes in T-lymphocyte subsets in peripheral blood and using them to assess immune response have aroused the people’s interests.

With the continuous development of minimally invasive surgery, more surgeons prefer laparoscopic surgery. It has been proven that laparoscopic surgery has the advantages of less trauma potential, faster postoperative recovery, and fewer complications [10]. Patients with poor basal immune status have more postoperative complications and more extended hospital stays [11]. Maintaining and improving patients’ immune status is important in perioperative management, yet relatively few reports have been published on the relationship between surgical approach and changes in patient immune status. Therefore, we considered 63 cases of patients with colorectal cancer admitted to the First Affiliated Hospital of Soochow University as the study subjects and analyzed the effect of different surgical procedures on the patients’ postoperative immune function.

## 2. Materials and Methods

### 2.1. Patient Data

**Figure 1:**
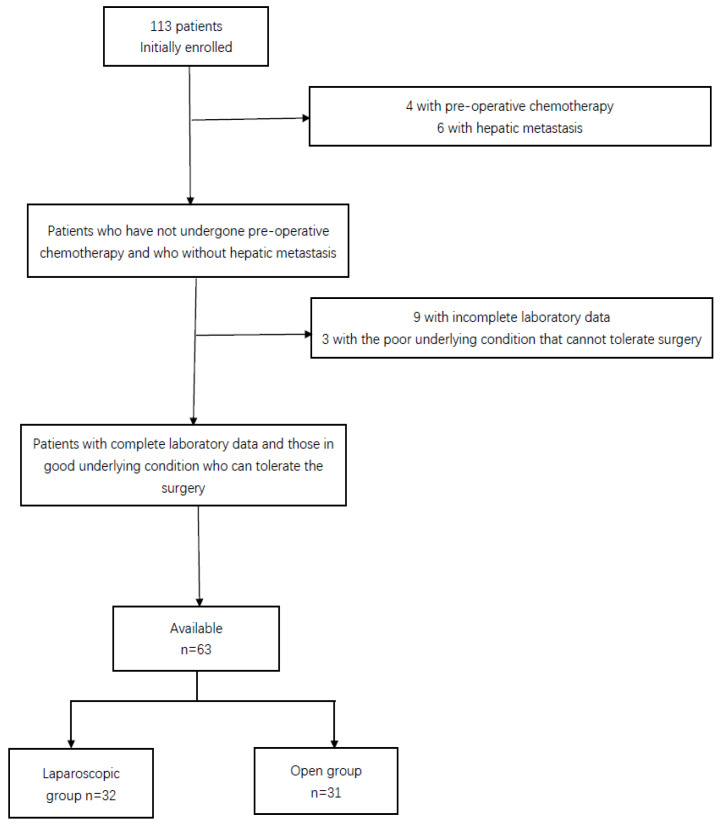
Filtering process of patient data from the initial inclusion of patients.

We conducted this study by the Declaration of Helsinki and obtained informed consent from all patients. This study was approved by the Ethics Committee of the First Affiliated Hospital of Soochow University (No. 421). The study population included 63 patients who were 18 years or older, had a body mass index (BMI) of 30 or less, had histologically proven colorectal adenocarcinoma with clinical stage II and III at the First Affiliated Hospital of Soochow University between 1 October 2021 and 31 December 2022 (Figure 1). These patients were prospectively included and had their peripheral blood levels of cellular immunity checked during treatment, and all patients had their disease diagnosed by colonoscopy and postoperative pathological biopsy. We excluded patients with a history of other malignant tumors such as cervical, uterine, or bladder; a medical history of Familial Adenomatosis Polyposis Coli, active Crohn’s disease, active ulcerative colitis; recent chemotherapy, radiotherapy, or endocrine therapy; the combination of distant metastases such as liver, lung, and bone; the complication of severe heart, lung, and kidney or due to hematologic disorders thus making them intolerant to surgery; psychiatric or addictive disorders that affected compliance to the protocol; conditions that would limit the success of laparoscopic resection such as multiple previous laparotomies or severe adhesions. After discussing the advantages and disadvantages of various surgical options with the surgeon, the patient chose laparoscopic surgery or open surgery. The patient was admitted to the hospital, the relevant tests were completed, and the patient was prepared for surgery. General anesthesia with tracheal intubation and routine urinary catheterization was used. Postoperative antibacterial drugs were routinely administered to prevent infection.

### 2.2. Immunological Index Acquisition

The general surgery nurse drew 5 mL of venous blood (15% Ethylene diamine tetraacetic acid tripotassium salt dihydrate anticoagulation) from a fasted, admitted patient at 7 am. Ethylene diamine tetraacetic acid anticoagulated blood was collected in 2 mL tubes. A total of 100 μL of anticoagulated whole blood was collected, 20 μL of CD45^+^/CD3^+^/CD4^+^/CD8^+^ cells was added, vortexed and mixed, incubated at room temperature and protected from light for 15 min. Then, 500 μL of cell lysate was added to each tube, vortexed and mixed, protected from light at room temperature for 10 min. Afterward, 2 mL of phosphate buffered saline was added to each tube, mixed, and detected by direct immunofluorescence labeling technique using flow cytometry for CD4^+^ T lymphocytes and CD8^+^ T lymphocytes to be detected by direct immunofluorescence labeling technique using flow cytometry; the percentages of CD4^+^ T cells and CD8^+^ T cells were recorded and the CD4^+^/CD8^+^ ratio was calculated. 

### 2.3. Statistical Analysis

All statistical analyses were performed by SPSS software (version 26.0). Measurement data are expressed as $\bar{X}$ ± S. Comparisons between groups were made using independent samples *t*-test, within-group comparisons before and after treatment were made using paired *t*-test and Pearson’s chi-square test, and *p* values less than 0.05 were considered significant. Logistic regression was used to eliminate the cofounders. R custom scripts (version 3.5.3) were used to generate all the figures and conducted the power analysis.

## 3. Results

### 3.1. Clinical Characteristics

In the Department of General Surgery of the First Affiliated Hospital of Soochow University, we enrolled 63 patients who underwent radical colorectal tumor surgery. All patients were treated according to the standard perioperative care protocol (a total of 31 patients underwent open surgery and 32 patients underwent laparoscopic surgery). Compared with the open group, patients in the laparoscopic group had significantly longer operative time (*p* < 0.05). There was no statistically significant difference in gender, age, BMI, blood type, hospitalization days, degree of tumor differentiation, tumor diameter, N stage, and tumor stage between the laparoscopic group and the open group (*p* > 0.05) (Table 1).

### 3.2. Difference of Immunological Indexes

Compared with the preoperative level, the postoperative number of CD3^+^ cells in the open group was not significantly different from that of the preoperative level, whereas the number of CD4^+^, CD8^+^, and CD4^+^/CD8^+^ ratio was significantly lower compared with the preoperative level; the postoperative number of CD3^+^ cells in the laparoscopic group was significantly higher compared with the preoperative level, whereas the number of CD4^+^ and CD8^+^ and the CD4^+^/CD8^+^ ratio were not significantly different compared with that of the preoperative level (Table 2).

**Figure 2:**
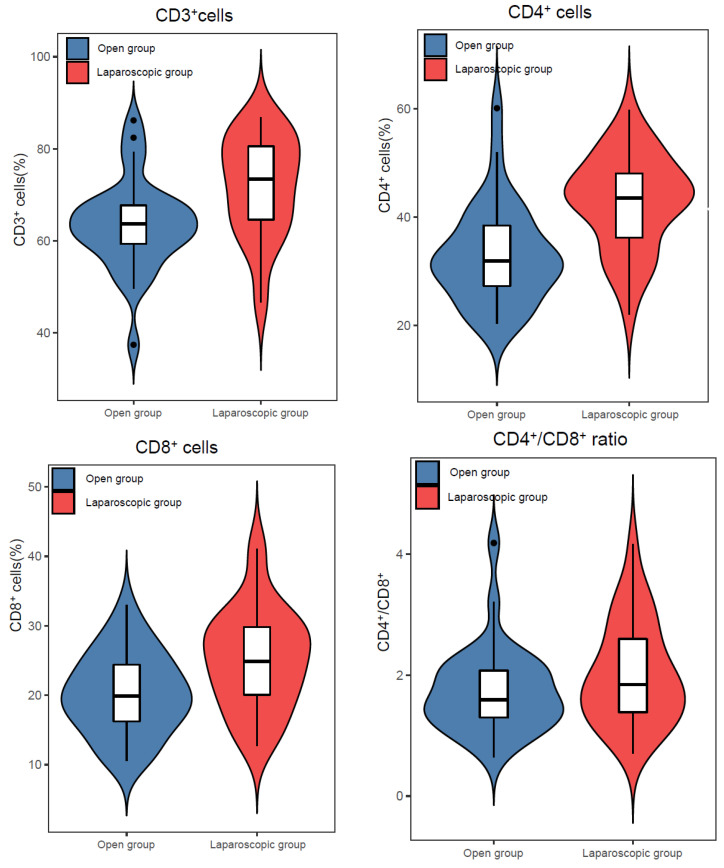
Changes in number of immune cells in open and laparoscopic groups at time point in post-surgery.

The number of CD3^+^, CD4^+^, CD8^+^ cells and the CD4^+^/CD8^+^ ratio in the laparoscopic group were not significantly different preoperatively compared to the open group, while they were significantly higher postoperatively compared to the open group (Table 2 and Figure 2).

The number of CD3^+^, CD4^+^, CD8^+^ cells and the CD4^+^/CD8^+^ ratio in the laparoscopic group were not significantly different from those in the open group before operation. In order to confirm that this is not the false negative caused by the low power, we conducted a power analysis, and calculated that the post hoc power of the number of CD3^+^, CD4^+^, CD8^+^ cells and the CD4^+^/CD8^+^ ratio were 80.46%, 80.12%, 87.89%, and 81.12%, respectively, which were greater than 80%. Based on this, we could confirm that the number of CD3 ^+^, CD4^+^, CD8^+^ cells and the CD4^+^/CD8^+^ ratio in the laparoscopic group did not differ from those of the open group before operation.

We also calculated that the post hoc power of the number of CD3^+^, CD4^+^, CD8^+^ cells and the CD4^+^/CD8^+^ ratio in the two groups of patients after surgery were 83.74%, 89.70%, 87.59%, and 40.82%, respectively. Although the post hoc power of the CD4^+^/CD8^+^ ratio was lower than 80%, our independent sample *t*-test still detected that there was a significant difference in the CD4^+^/CD8^+^ ratio between the two groups of patients after operation.

### 3.3. Analysis of Influencing Factors

The results of univariate logistic regression analysis showed that the effects of age, tumor location, and types of surgery on patients’ postoperative CD3^+^ T cells were statistically significant. Then, the effect of age and tumor location was excluded by multivariate logistic regression analysis, and it was determined that the effect of types of surgery on patients’ postoperative CD3^+^ T cells remained statistically significant (Table 3). Laparoscopic-assisted surgery can reduce the inhibition of immune functions compared with open surgery.

Similarly, after excluding the effect of operation time, tumor stage as well as tumor location by multivariate logistic regression analysis, the effect of types of surgery on patients’ postoperative CD4^+^T and CD8^+^T remained statistically significant (Table 4 and Table 5). These findings suggested that laparoscopic-assisted surgery can reduce the inhibition of immune functions compared with open surgery. However, the results of both univariate logistic regression and multivariate logistic regression analysis showed that the surgical approach of patients had no statistically significant influence on the CD4^+^T/CD8^+^T ratio of patients after surgery (Table 6).

## 4. Discussion

It is well known that the stress response induced by surgical trauma affects the immune system and postoperative immunosuppression; it can also make CD3^+^ and CD4^+^ cell counts and CD4^+^/CD8^+^ ratios decrease [9]. The main immune mechanism against tumors is cellular immunity, which directly reflects anti-tumor activity. Therefore, avoiding suppression of cellular immunity plays an important role in prognosis of colon cancer surgery [12]. Surgery, whether laparoscopic or open, is a controlled trauma that can trigger changes in inflammation, neuroendocrine and immune function. With the advent of laparoscopic surgery, the ability to enter the patient’s abdominal cavity through small openings, carefully segment and repair tissue and reduce the risk of bleeding has been greatly enhanced. Laparoscopic radical colorectal cancer surgery is a safe and effective surgical method, and its advantages include the following: (1) the surgical field of view is wide and has a magnifying effect, which can determine the intra-abdominal tissues and lesions; (2) the operation is delicate and gentle, and the interference with the internal organs of the abdominal cavity is small; (3) the operation is less invasive, causes less bleeding, pain, implies less adhesive intestinal obstruction, and fewer postoperative complications and faster recovery [10]; (4) less stress on the patient’s organism and less impact on cell-mediated immunity [13].

Konstantinos E. and colleagues reportedly studied the acute phase response after open and laparoscopic surgery. Their seminal report compared interleukin- 6 (IL-6), tumor necrosis factor-α, c-reactive protein (CRP), Toll-like receptors-2 and Toll-like receptors-4 levels. They concluded that the inflammatory response and resulting stress response after laparoscopic surgery were significantly lower than in patients undergoing open surgery, which has a clear short-term clinical benefit for patients [14]. Mauro P. reviewed the early postoperative and oncological outcomes after laparoscopic colectomy for T4 cancer compared with open surgery, determining that laparoscopic colectomy for T4 colonic cancer is safe and is associated with better clinical outcomes than open surgery and similar oncological outcomes. Mauro’ s research demonstrates that in regard to long-term clinical benefits, laparoscopic surgery is better for patients than open surgery [15].

The development of malignant tumors is closely related to the immune function, affected by the factors such as surgery, trauma, and infection. The immune system mainly includes cell-mediated immunity and humoral immunity. It is believed that cell-mediated immunity is the mainstay of anti-tumor immunity, while humoral immunity only plays a synergistic role in some cases, and some cytokines are also involved in the body’s immune response [16]. T-lymphocyte-mediated cell-mediated immunity is involved in the postoperative immune response. CD3^+^ T-cells are the main marker of mature T-lymphocytes in peripheral blood, representing the overall level of cell-mediated immunity. Human mature T-lymphocytes are divided into CD4^+^ and CD8^+^ T-cells depending on their phenotypes. CD4^+^ T-cells are helper T-cells, which have helper functions and are co-receptors for T cell receptor signaling, and upon activation can release a large number of cytokines, enhancing the antitumor effect. Apart from producing cytokines, different subsets of CD4^+^ T cell has been identified, including cytotoxic CD4^+^ T cells, which possess cytotoxic programs and can directly kill cancer cells [17].CD8^+^ T cells are cytotoxic and suppressive T cells that are involved in the maturation and positive selection of restrictive cytotoxic T lymphocyte for major histocompatibility complex-I [18]. In recent studies, multiple subsets of CD8^+^ T cells have been detected in tumor microenvironments, called Tc subsets, each with distinct effector functions and cytotoxic potential, possibly influencing the antitumor response and patient outcomes [19]. The CD4^+^/CD8^+^ ratio is approximately 1.2–2.0, which is an important indicator of the body’s immune homeostasis. Decrease in this ratio often indicates immune dysfunction, and a significant reduction or inversion is often used as an indicator of severe disease and poor prognosis [20].

Previously, studies on the effects of laparoscopic and open surgery on the immune system have focused on IL-6 and CRP [21]. The IL-6 promotes tumor angiogenesis and reduces inter-tumor cell adherence; it inhibits the body’s anti-tumor immunity; it also has anti-apoptotic effects, thus promoting tumorigenesis. IL-6 plays an important role in the metastasis and progression of colorectal cancer [9]. CRP is a more sensitive inflammatory response protein produced by hepatocytes induced by IL-6, and its expression level increases when the body is exposed to trauma or infection [22]. Surgery remains the mainstay of treatment for colorectal cancer; however, it also leads to transient immunosuppression and diminished tumor resistance. Experimental animal studies have shown that immunity is better preserved after laparoscopic colorectal surgery, and open surgery is associated with accelerated tumor growth compared to laparoscopic surgery [23].

The application of pneumoperitoneum requires the introduction of large amounts of CO_2_ gas into the abdominal cavity, and studies have shown that CO_2_ pneumoperitoneum may produce hypercapnia and have immunological effects on the body [24]. Kim I. and his colleague reported that low intra-abdominal pressure during laparoscopic colorectal surgery, which means less CO_2_, preserved innated immune homeostasis and formed a valuable addition to future enhanced recovery [25]. This issue has been controversial; however, from the present study, even though pneumoperitoneum affects the immune function of the organism, its effect is less compared to an open abdominal injury. In general, laparoscopic surgery causes less loss of immune function of the organism compared to open surgery, for better short-term postoperative benefit.

To conclude, we conducted studies on immune function parameters and the results showed that the number of CD3^+^ cells in the open group, compared with the preoperative level, was not significantly different from that before surgery, while the number of CD4^+^ and CD8^+^ cells and the CD4^+^/CD8^+^ ratio was significantly lower. The number of CD3^+^ cells in the laparoscopic group was significantly higher than that before surgery, while the number of CD4^+^, CD8^+^ cells and CD4^+^/CD8^+^ ratio was not significantly different from those before surgery. Compared with the open group, there was no significant difference in the number of CD3^+^, CD4^+^, CD8^+^ cells and the ratio of CD4^+^/CD8^+^ in the laparoscopic group before surgery, but the number was significantly higher than in the open group after surgery. In Gang Wang’s study, it was suggested that laparoscopic colon surgery effectively protected postoperative cellular immunity, and the decrease in the number of CD3^+^ and CD4^+^ cells and the CD4^+^/CD8^+^ ratio was significantly attenuated compared with open surgery patients. This is consistent with our findings [9]. However, Wichmann and Tang reported no difference in the number of CD3^+^ and CD4^+^ cells after laparoscopic surgery compared with open surgery, and only a small difference in the number of compliments, but the difference may exist due to the significantly longer surgery time in the laparoscopic group compared with the open group, thus weakening the function of laparoscopic surgery in reducing immunosuppression because of the excessive surgery time [21,26]. In the surgical treatment of lung cancer, Lian-Bin Zhang et al. determined that the postoperative T-lymphocyte subpopulation cell count was significantly higher in patients in the video-assisted thoracic surgery group compared to the traditional open surgery group, suggesting that the video-assisted thoracic surgery lowers the postoperative acute phase response and reduces immune suppression [27]. Li-Wen Zhou et al. detected higher postoperative CD4^+^ and CD8^+^ cell counts in patients on tramadol compared to those operated on without tramadol, which may be due to the perioperative patient’s pain-mediated immunosuppression and consequent decrease in immune cell counts, which is also consistent with our findings [28]. Laparoscopic surgery has been shown to have a lower incidence of postoperative pain than open surgery [29], and thus patients undergoing laparoscopic surgery may benefit in terms of immune function.

This study is one of the few reports in which CD4^+^ and CD8^+^ cell counts were detected in peripheral blood after many recent studies investigating the relationship between T lymphocyte subsets in tumor microenvironment and tumor development as well as prognosis [30]. However, there are several drawbacks, and although we detected significantly higher numbers of CD4^+^ and CD8^+^ cells in the laparoscopic group than in the open group, the reasons for this occurrence cannot be well explained because laparoscopic surgery requires filling the peritoneal cavity with a large amount of CO_2_ gas, and West studied cytokine production in peritoneal macrophages incubated in CO_2_. Macrophage tumor necrosis factor and interleukin-1 responses to bacterial endotoxin were lower in macrophages incubated in CO_2_ than in macrophages incubated in air or helium. West hypothesized that impairment of peritoneal macrophage cytokine production may contribute to the apparent lack of inflammatory systemic response during laparoscopic surgery [31]. We speculate that this may also apply to explain the changes in CD4^+^ and CD8^+^ cell numbers during laparoscopic surgery.

Our study has a few limitations. Firstly, we had a relatively small number of patients previously tested for T lymphocytes and the numerous exclusion criteria, and we have now made the detection of T lymphocyte counts in colorectal cancer patients a routine test. We will collect more patient data in the near future to draw more convincing conclusions. Secondly, the blood biomarkers analyzed in this study were nonspecific and may be influenced by various physiological or pathological factors. In addition, there are many blood indicators that can reflect the changes in immune status of patients. IL-6, CRP, reactive oxygen species, superoxide dismutase, etc., have been reported in the literature and are also closely related to the immune function of patients [32,33,34]. Thus, we subsequently plan to increase the tests in collaboration with clinical laboratory and also to verify them at the tissue level to support our conclusions. In addition, we employed the statistical technique of power analysis, but this power is no longer important because the results have been obtained. Post hoc power calculations were based on the observed effect entirely, but the lack of statistical power may substantially affect the size and even the direction of the observed effect. Finally, because of the lack of long-term prognostic data, we cannot yet determine the impact of the reduction in immunosuppression by laparoscopy on the long-term prognosis of patients, but studies have shown that this advantage of laparoscopy is valuable for the long-term survival of patients [35], and we will continue to follow up this cohort of patients to study the long-term impact of this surgical approach.

There is no doubt that the clinical efficacy of laparoscopic surgery has been established [36] and that the systemic immune impact of laparoscopic surgery may be even lesser [37]. With increased research at the cellular and molecular levels, the systemic, metabolic, and immune effects of laparoscopic surgery will be better understood and patients will hopefully benefit from it.

In conclusion, this study determined that laparoscopic-assisted surgery can reduce the inhibition of immune functions compared with open surgery. It is clear that laparoscopic surgery is known to provide an immunological advantage, but whether it provides a survival advantage needs further study.

## Figures and Tables

**Table 1:** General material comparison in perioperative period of the open and laparoscopic groups (x ± s).

Items	Open Group (n = 31)	Laparoscopic Group (n = 32)	*p*
Gender			0.677+
Male	20	19	
Female	11	13	
Age (years)	68.58 ± 9.135	64.97 ± 8.102	0.102
BMI (kg/m^2^)	22.17 ± 2.43	22.49 ± 5.14	0.746
Operation times (min)	156.32 ± 48.10	188.78 ± 65.10	*0.028*
Postoperative hospital stay (days)	12.46 ± 2.41	12.50 ± 3.99	0.753
Tumor differentiation			0.892+
Well or Moderate	14	15	
Poorly	17	17	
Maximal tumor diameter (cm)			0.246+
≤5 cm	19	24	
>5 cm	12	8	
N stage			0.267+
0	18	14	
1	6	12	
2	7	6	
Tumor stage			0.378+
II	18	15	
III	13	17	
Blood Type			0.742+
A	10	10	
B	9	12	
O	7	6	
AB	5	4	
Tumor Location			0.367+
Right colon cancer	7	11	
Left colon cancer	10	9	
Rectal cancer	14	12	

*p*-values were estimated by *t*-test; + *p*-values were estimated by Pearson’s chi-square test; *p* < 0.05 are highlighted in bold italic.

**Table 2:** Changes in number of immune cells in open and laparoscopic groups at different time point in pre- and post-surgery (x ± s).

Group	CD3^+^T (%)	CD4^+^T (%)	CD8^+^T (%)	CD4^+^T/CD8^+^T
Open group				
The day before operation	66.59 ± 11.05	42.35 ± 9.21	23.08 ± 8.40	2.04 ± 0.78
The third day after operation	63.56 ± 9.72 *	32.86 ± 9.02 *#	20.21 ± 5.80 *#	1.73 ± 0.71 *#
Laparoscopic group				
The day before operation	69.10 ± 10.70	43.76 ± 9.64	24.52 ± 8.13	2.06 ± 1.17
The third day after operation	71.87 ± 10.89 #	42.75 ± 8.70	25.08 ± 7.20	2.01 ± 0.85

* The comparison in laparoscopic group at the same time point, *p* < 0. 05. # The comparison in the same group on the day before operation, *p* < 0. 05.

**Table 3:** Logistic regression analysis of the patient’s postoperative CD3^+^T.

Items	Postoperative CD3^+^T			
	Univariate Analysis		Multivariate Analysis	
	OR (95%CI)	*p*	OR (95%CI)	*p*
Gender (male/female)	1.379 (0.497~3.825)	0.537		
Age (years, ≤65/>65)	0.930 (0.873~0.991)	0.025	0.933 (0.870~1.001)	0.052
BMI (kg/m^2^, ≤18.5/>18.5)	1.027 (0.906~1.165)	0.676		
Operation times (min, ≤150/>150)	1.003 (0.994~1.011)	0.544		
Tumor differentiation (Well or Moderate/Poor)	1.071 (0.398~2.887)	0.891		
Maximal tumor diameter (≤5 cm/>5 cm)	1.406 (0.484~4.079)	0.531		
Tumor stage (II/III)	0.941 (0.350~2.531)	0.904		
Blood type				
AB	1			
A	2.321 (0.467~11.545)	0.303		
B	0.769 (0.158~3.744)	0.745		
O	1.458 (0.264~8.048)	0.665		
Tumor location				
Rectal cancer	1			
Right colon cancer	4.911 (1.325~18.205)	0.017	5.349 (1.250~22.898)	0.024
Left colon cancer	2.099 (0.626~7.037)	0.230		
Types of surgery (open/laparoscopic)	4.620 (1.599~13.349)	0.005	3.908 (1.225~12.468)	0.021

**Table 4:** Logistic regression analysis of the patient’s postoperative CD4^+^T.

Items	Postoperative CD4^+^T			
	Univariate Analysis		Multivariate Analysis	
	OR (95%CI)	*p*	OR (95%CI)	*p*
Gender (male/female)	0.612 (0.219~1.710)	0.349		
Age (years, ≤65/>65)	0.959 (0.903~1.017)	0.161		
BMI (kg/m^2^, ≤18.5/>18.5)	0.897 (0.771~1.042)	0.155		
Operation times (min, ≤150/>150)	1.012 (1.002~1.023)	0.021	1.012 (0.999~1.024)	0.064
Tumor differentiation (Well or Moderate/Poor)	0.830 (0.308~2.237)	0.712		
Maximal tumor diameter (≤5 cm/>5 cm)	1.895 (0.645~5.569)	0.245		
Tumor stage (II/III)	0.376 (0.136~1.043)	0.060		
Blood type				
AB	1			
A	0.533 (0.109~2.616)	0.438		
B	1.067 (0.221~5.145)	0.936		
O	0.933 (0.169~5.151)	0.937		
Tumor location				
Rectal cancer	1			
Right colon cancer	7.875 (1.964~31.574)	0.004	10.384 (2.076~51.936)	0.004
Left colon cancer	2.5 (0.733~8.524)	0.143		
Types of surgery (open/laparoscopic)	6.247 (2.093~18.641)	0.001	5.656 (1.602~19.982)	0.007

**Table 5:** Logistic regression analysis of the patient’s postoperative CD8^+^T.

Items	Postoperative CD8^+^T			
	Univariate Analysis		Multivariate Analysis	
	OR (95%CI)	*p*	OR (95%CI)	*p*
Gender (male/female)	1.379 (0.497~3.825)	0.537		
Age (years, ≤65/>65)	0.998 (0.942~1.056)	0.934		
BMI (kg/m^2^, ≤18.5/>18.5)	1.012 (0.894~1.146)	0.848		
Operation times (min, ≤150/>150)	0.997 (0.988~1.005)	0.423		
Tumor differentiation (Well or Moderate/Poor)	2.338 (0.848~6.447)	0.101		
Maximal tumor diameter (≤5 cm/>5 cm)	0.711 (0.245~2.065)	0.531		
Tumor stage (II/III)	1.775 (0.654~4.819)	0.260		
Blood type				
AB	1			
A	1.250 (0.257~6.070)	0.782		
B	2.031 (0.417~9.886)	0.380		
O	0.781 (0.139~4.387)	0.779		
Tumor location				
Rectal cancer	1			
Right colon cancer	0.500 (0.144~1.737)	0.275	0.353 (0.090~1.389)	0.136
Left colon cancer	2.167 (0.630~7.454)	0.220		
Types of surgery (open/laparoscopic)	3.471 (1.232~9.782)	0.019	4.780 (1.495~15.280)	0.008

**Table 6:** Logistic regression analysis of the patient’s postoperative CD4^+^T/CD8^+^T.

Items	Postoperative CD4^+^T/CD8^+^T			
	Univariate Analysis		Multivariate Analysis	
	OR (95%CI)	*p*	OR (95%CI)	*p*
Gender (male/female)	1.379 (0.497~3.825)	0.537		
Age (years, ≤65/>65)	0.968 (0.913~1.026)	0.275		
BMI (kg/m^2^, ≤18.5/>18.5)	1.006 (0.889~1.139)	0.921		
Operation times (time, ≤150/>150)	1.017 (1.005~1.028)	0.005	1.016 (1.004~1.028)	0.009
Tumor differentiation (Well or Moderate/Poorly)	1.071 (0.398~2.887)	0.891		
Maximal tumor diameter (≤5 cm/>5 cm)	0.711 (0.245~2.065)	0.531		
Tumor stage (II/III)	1.214 (0.451~3.269)	0.701		
Blood type				
AB	1			
A	0.269 (0.051~1.420)	0.122		
B	0.813 (0.157~4.197)	0.804		
O	0.429 (0.073~2.500)	0.346		
Tumor location				
Rectal cancer	1			
Right colon cancer	3.545 (0.974~12.905)	0.055		
Left colon cancer	0.992 (0.299~3.285)	0.989		
Types of surgery (open/laparoscopic)	2.024 (0.742~5.519)	0.168	1.360 (0.451~4.102)	0.586

## Data Availability

The data supporting the findings of this study are available from the corresponding author upon reasonable request.
